# Next-Generation Technologies and Systems Biology for the Design of Novel Vaccines Against Apicomplexan Parasites

**DOI:** 10.3389/fvets.2021.800361

**Published:** 2022-01-07

**Authors:** Mariela Luján Tomazic, Virginia Marugan-Hernandez, Anabel Elisa Rodriguez

**Affiliations:** ^1^Instituto de Patobiología Veterinaria (IPVET), INTA-CONICET, Hurlingham, Argentina; ^2^Universidad de Buenos Aires, Facultad de Farmacia y Bioquímica, Catedra de Biotecnología, Ciudad Autónoma de Buenos Aires, Argentina; ^3^The Royal Veterinary College, University of London, London, United Kingdom

**Keywords:** apicomplexa, CRISPR/Cas9, omics, systems biology, vaccines

## Abstract

Parasites of the phylum Apicomplexa are the causative agents of important diseases such as malaria, toxoplasmosis or cryptosporidiosis in humans, and babesiosis and coccidiosis in animals. Whereas the first human recombinant vaccine against malaria has been approved and recently recommended for wide administration by the WHO, most other zoonotic parasitic diseases lack of appropriate immunoprophylaxis. Sequencing technologies, bioinformatics, and statistics, have opened the “omics” era into apicomplexan parasites, which has led to the development of systems biology, a recent field that can significantly contribute to more rational design for new vaccines. The discovery of novel antigens by classical approaches is slow and limited to very few antigens identified and analyzed by each study. High throughput approaches based on the expansion of the “omics”, mainly genomics and transcriptomics have facilitated the functional annotation of the genome for many of these parasites, improving significantly the understanding of the parasite biology, interactions with the host, as well as virulence and host immune response. Developments in genetic manipulation in apicomplexan parasites have also contributed to the discovery of new potential vaccine targets. The present minireview does a comprehensive summary of advances in “omics”, CRISPR/Cas9 technologies, and in systems biology approaches applied to apicomplexan parasites of economic and zoonotic importance, highlighting their potential of the holistic view in vaccine development.

## Introduction

Apicomplexans parasites are a major cause of disease in humans and animals worldwide. These pathogenic unicellular microorganisms can be zoonotic, threatening human populations, and/or compromise animal health and welfare, causing an economic impact to the farming industry. The most relevant human parasite, *Plasmodium* spp., is the etiological agent of malaria in humans. Other zoonotic apicomplexans are the cyst forming *Toxoplasma gondii* that is the model organism for research within the phylum, and *Cryptosporidium parvum*. Parasites of this phylum also encompass several species causing diseases with welfare and economic impact in livestock and poultry, as well as in wild animals and pets (1), some examples are babesiosis, eimeriosis (coccidiosis), neosporosis, besnoitiosis, and theileriosis, among others, caused by species from the genera *Babesia, Eimeria, Neospora, Besnoitia, and Theileria*, respectively. Although there are notable differences between apicomplexans, they are obligate intracellular microorganisms for some stages, with complex lifecycles. Typically, parasites of this phylum contain an apical complex composed of the conoid and specialized secretory organelles that includes micronemes, rhoptries, and dense granules, which are involved in host cell attachment, invasion, and the establishment of an intracellular parasitophorous vacuole within the host cell (2); proteins secreted by these organelles has been classical candidates for vaccine development (3).

The advanced molecular tools for the study of some of these organisms can provide detailed information of relevant biological processes that can be an invaluable source for antigen discovery and vaccine development. Despite that the first vaccines were developed in humans in 1796, with the smallpox vaccine (4), no vaccines were developed in the veterinary field until a centenary later, being pioneer the vaccine against chicken cholera developed in 1879 (5) and a few years later against bovine babesiosis developed by Pound et al. (6), and Connaway et al. (7), which were followed by vaccines against avian coccidiosis (8) developed in the 1950s. However, the major contribution to the development of vaccines against parasites came from the efforts to combat malaria in the 1960s (9). Besides all the progress made towards the control of human malaria disease, between 2010 and 2017 the incidence has only been reduced by an 18% (10); and despite its importance in public health, there is only one vaccine approved by the European Medicines Agency against human malaria: Mosquirix (RTS,S– GlaxoSmithKline) (11, 12) that has recently been recommended by the World Health Organization (WHO) for its widespread use in children in sub-Saharian Africa and other regions with moderate to high risk of *Plasmodium falciparum* transmission (13). To date, only a small number of vaccines against apicomplexan parasites of veterinary importance are available (14). The main barriers in protozoal vaccinology relate to their complex and multifaceted lifecycles, presenting different stages and intricate interactions with the host, not yet well understood, impairing the development of successful vaccines.

The discovery of novel antigens by classical approaches is slow and limited to very few antigens (15). High-throughput technologies based on the expansion of the “omics” (defined as the ‘characterization and quantification of pools of biological molecules' such DNA—genomics, RNA—transcriptomics, proteins—proteomics, metabolites—metabolomics, lipids—lipidomics) has facilitated the functional annotation of the genomes for many of these parasites, significantly improving the understanding of their biology, interactions with the host (16), as well as providing novel targets for vaccine development (17). Additionally, advances in genetic manipulation of parasites have allowed the generation of transgenic populations to understand biological processes (18) or experimentally validate the gene function (19).

**Figure 1:**
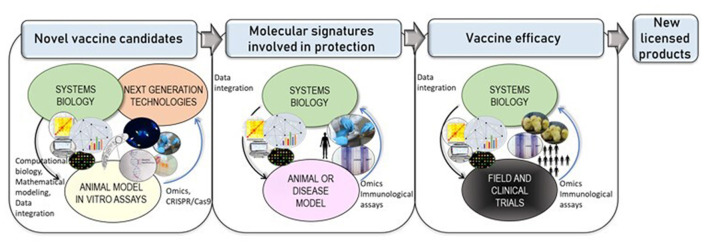
Next-generation technologies and systems biology leading to novel vaccines. Cutting-edge technologies such as comparative “omics” or CRISPR/Cas9-based technologies allow functional characterization of genes with an hypothetical annotation. Systems biology approaches allow positive/negative dynamic feedback in an iterative process (indicated with blue arrows), by combining the use of data generated by molecular biology and high-throughput technologies with computational biology and mathematical modeling, have enabled the study of host immune responses and host-parasite interactions that could lead to the discovery of novel vaccine targets. Furthermore, systems biology applied to animal or disease model holds to the discovery of host immune signatures. Finally, upon vaccine design and experimental evaluation vaccines are evaluated under different formulations and regimens in animal/human models in controlled *in vivo* trials–i.e. field or clinical -, systems-based approaches will integrate immunological, experimental and “omics” data, assessing vaccine efficacy in the specific target populations, supporting further improvements and leading to new licensed products for apicomplexan parasites.

Systems biology is defined as an interdisciplinary approach within the area of biomedical research that combines big data derived from multi-omic studies with computational and statistical analysis, aimed to unravel interactions and dynamics from single to complex biological levels (20, 21). The use of data generated by high-throughput-omics technologies in the context of vaccination has raised the new field of “systems vaccinology”, also known as vaccinomics, aimed to understand the biological processes involved in vaccine-induced immunity as a holistic view (22). Systems biology-based approaches can improve the understanding of protective immune predicting behavior of the immune system in responses after vaccination. Given the current gaps in host-parasite interactions and the need for novel vaccine candidates, systems-biology could also help to fill-up the current empties in knowledge, allowing the discovery of potential novel targets (23) for chemoprophylaxis, vaccine development and valuable surrogate markers (24) as depicts Figure 1. The following section summarizes the advances in “omics”, CRISPR/Cas9 technologies, and the latest findings in this field of systems biology for the most relevant apicomplexan parasites.

## Next-Generation Technologies

### Omics

Apicomplexan parasites have large genomes with complex proteomes (25), of which most proteins remain uncharacterized (26). One of the first sequenced apicomplexan genomes was from *P. falciparum* in 2002, this has enabled a better understanding of its lifecycle, and has highlighted that a large proportion of genes are devoted to immune evasion and host-parasite interactions (25). This has also provided a new starting point for future studies focused on surveillance strategies and vaccine development. The genome annotation of *Eimeria* species has revealed that these parasites possess the most repeat-rich proteomes ever described, as well as the presence of retrotransposons-like elements. Analysis of *Eimeria* genes involved in basic biological functions and host-parasite interaction highlights adaptations to a relatively simple developmental lifecycle and a complex array of co-expressed surface proteins involved in host cell binding (27). Annotation of genomic databases can have direct application on the identification of vaccine candidates, and in this way, genetic fingerprinting has allowed the identification of the immunoprotective antigens AMA1 and IMP1 in *Eimeria maxima* (28).

The transcriptome of *C. parvum* has revealed metabolic features associated with environmental survival and stresses (29). Similarly, the analysis of *T. gondii* transcriptome has identified developmentally regulated genes including surface proteins (a SAG1-related protein, SRS9, and a mucin-domain containing protein), regulatory and metabolic enzymes (methionine aminopeptidase, oligopeptidase, aminotransferase, and glucose-6-phosphate dehydrogenase homologs), and a subset of genes encoding proteins from secretory organelles (MIC1, ROP1, ROP2, ROP4, GRA1, GRA5, and GRA8), many of which have become important vaccine candidates (30).

Initial transcriptomic studies based on microarrays, “expressed sequence tag” (EST) collections, “serial analysis of gene expression” (SAGE), and massively parallel signature sequencing (MPSS) had several limitations, such as the restricted dynamic range and sample preparation, unable to be simultaneously processed and analyzed for both host and pathogen. Current RNA sequencing (RNA-seq), a breakthrough molecular tool, can now provide the full transcript profile (transcriptome) of cellular RNA with a large dynamic range and improved sensitivity (31). Transcriptomic studies are widely used for diverse purposes. For example, global transcriptome analysis for both host and *T. gondii* during the establishment of chronic infection in mice was performed by Pittman and collaborators (2014) (32). The results demonstrated the influence of parasite development on host gene transcription as well as the influence of the host environment on parasite gene transcription. Importantly, the host genes associated with the immune response were more abundant during the chronic infection than in the acute phase. Conversely, parasite genes that are highly expressed in both acute and chronic infection were involved in transcription and translation, highlighting that both stages of the parasites can actively synthesize proteins.

Comparative transcriptomics can contribute to identifying biomarkers of resistance to parasite infection, enabling a better understanding of the onset of the immune response such as was demonstrated by Bremmer and collaborators (2019) (33) in two lines of chicken with distinct resistance or susceptibility to *E. maxima* infection. They found that the timing at which the immune response is mounted is paramount to resistance, in particular for early induction of IFN-γ and IL-10, with a new gene identified (*IL-21*) associated to resistance to *E. maxima* infection.

Proteomics represents a step forward for the understanding of the actual proteins expressed by these genomes in specific conditions, and how well the current models can predict protein sequence. Proteomics data can supplement genome annotation efforts, by confirming gene models or correcting gene annotation errors (34). In order to improve the proteomic understanding of gene expression in these protozoa parasites, large-scale proteomic studies have been undertaken in *P. falciparum* (35, 36), *C. parvum* (37, 38), and *T. gondii* (39) as well as other studies of sub-proteomes (40, 41). The comparative study of the proteome of isolates of *Neospora caninum* exhibiting different virulence levels led to the identification of novel immunoprophylactic targets (42) that have been evaluated as vaccine candidates with good results in immunoprotection (43). A recent multiplexed proteomic study has demonstrated significant modulations in key physiological pathways, such as lipid metabolism, cytokine signaling, complement, and coagulation cascades in severe malaria, providing blood markers that could improve monitoring the disease progression (44).

In addition to proteomics (39), lipidomics analysis in *T. gondii* has been addressed in the last decade, revealing parasite-specific proteins and lipids, unraveling parasite-host interactions (45). Recently, Kadesch and collaborators (2020) (46) carried out a mass spectrometry imaging in *Besnoitia besnoiti* and *T. gondii* infection in primary bovine umbilical vein endothelial cells using atmospheric-pressure scanning microprobe matrix-assisted laser desorption/ionization (AP-SMALDI) mass spectrometry imaging (MSI), an emerging technique that provides high resolution and allows analysis of single cells, allowing a metabolomic characterization. This study has revealed biomolecular markers of infection in both parasites and has shown striking differences in the metabolites during infection between both parasites, despite their closer phylogenic relationship, related in particular to lipid classes such as phosphatidylcholines, phosphatidylethanolamines, phosphatidylglycerols, cardiolipins, phosphatidic acids, and phosphatidylinositol (46).

### Clustered Regularly Interspaced Short Palindromic Repeats/Associated Protein 9 (CRISPR/Cas9) Systems

There is a wide range of emerging tools adapted now to many Apicomplexa that can allow the discovery of antigens with potential interest as vaccine targets. Next-generation technologies of gene editing such as CRISPR/Cas9 have contributed to this issue, moving forward gene functional studies, by knocking-out, repressing, activating, or tagging genes in species where it was not possible before by other methods. This has enhanced and speed-up the understanding of the biology of these parasites, providing newly characterized genes as potential targets for vaccine development (Figure 1). It has revolutionized parasitology research (47), being successfully applied to *P. falciparum* (48, 49) in the first place, and then to other apicomplexan parasites (*T. gondii, Plasmodium yoelii, C. parvum*, and *Eimeria* spp.) (19, 50–53). For *C. parvum* (54) step-by-step protocols have been published, becoming an invaluable resource for the research community. Improved CRISPR/Cas9 based technologies or the generation of knock-out libraries in *T. gondii* have allowed the functional characterization of a greater number of genes related to virulence (55), leading to novel therapeutic targets. Recently, a CRISPR/Cas9 strategy coupled with glycomics in *T. gondii* has allowed new insights into the role of glycogens, and the discovery of novel genes (56). Glycans are involved in many cellular functions such as invasion, oxygen sensing, wall formation of parasite stages (cyst), and nutrient storage; hence, this study may provide knowledge regarding key cellular functions and also regarding virulence in related parasites.

## Systems Biology Applied to Apicomplexa

### Systems Immunology

System biology-based approaches have been extensively applied to studies of host-parasite interactions (23) and were recently reviewed by Swann and collaborators (2015) (26). Systems biology-based approaches applied to the study, analysis, and understanding of the immune system are also known as “systems immunology”, and has been applied to identify immune signatures upon infection of *Plasmodium* sp. (57–60) and *T. gondii* (61). The analysis of transcriptomic data from both, parasite and host, has revealed some new aspects of parasite immunology and are summarized in Table 1. A recent study carried out using blood from people with uncomplicated *P. falciparum* malaria indicated that the innate immune response, cytokines (IL-1β, IL-6, TNF-α, and IFN-γ), and apoptosis pathways were acutely upregulated in the group under study with concomitant downregulation of immune-modulatory and apoptosis inhibitory genes (58). These results are in contrast with a previous report from Ockenhouse and collaborators (2006) (57) that showed that genes involved in phagocytosis and inflammation, including the cytokines TNF-α, IFN-γ, and IL-1β were downregulated. The results and the different interpretations that raise from the different datasets may be affected by variations in the population under study, clinical phenotype, and vaccine regimen. Likewise, due to the high degree of heterogeneity between biological samples, high variability is expected. Additionally, immune responses are affected by other factors such as age, genetics, stress, comorbidity, that hinder the interpretation of the immune mechanisms. Therefore, the more data is collected the more hypothesis would arise and novel findings and biomarkers will be generated, clarifying this issue.

**Table 1:** Systems biology approaches in the field of vaccinology and immunology applied to *P. falciparum* and *T. gondii*.

**Field**	**Species**	**Host or target population**	**Disease**	**Finding**	**Methods**	**Reference**
Immunology	*P. falciparum* ^1^	Human	Malaria	Immune signatures: upregulation of genes of the innate response; downregulation of genes involved in phagocytosis and inflammation. Differences in apoptosis genes between symptomatics/ presymtomatics, or uncomplicated malaria	Microarray, computational approaches	(57, 58)
				Immune markers that correlates with severity. Genetic variation associated with severe malaria symptoms and drug-resistance	RNA-seq, computational approaches	(59)
	*Plasmodium ashfordi*	Birds, Mice	Malaria	Genes differentially expressed and different T- cell activation with parasitemia stages	RNA-seq, gene set enrichment analysis	(60)
						
	*T. gondii*	Pigs	Toxoplasmosis	Parasite actively regulates host genes related to the immune responses between acute and chronic infection	Transcriptomics, gene set enrichment analysis	(61)
Vaccinology	*P. falciparum* ^1^	RTS,S vaccinated volunteers	Human malaria	Up-regulation of genes associated adaptive response. Possible innate genes markers of protection	Transcriptomics, gene enrichment analysis, predictive modeling	(62, 63)
		CSP^2^ vaccinated volunteers	Human malaria	Molecular signatures of protective immunity. Differential expression of genes of immune response, protein synthesis, and mitochondrial processes in protected and non–protected individuals	Gene array, predictive modeling. RNA-seq, module correlation network analysis, immunological methods	(64, 65)

1 *Most recent studies, for further information refer to Tran & Crompton 2019 (66)*.

2 *CSP: immunization with live sporozoites*.

Yamagishi and collaborators (2014) have performed a transcriptomic analysis both in *P. falciparum* and humans to elucidate the mechanism of host interactions. This work has identified human and parasite genes and pathways that correlated with various clinical data (Table 1), providing novel targets for therapy. Furthermore, it has been identified genetic variations in anti-malaria drug resistance-related genes as well as associated with severe malaria symptoms (59). Another transcriptomic analysis focused on avian malaria has revealed differences in the sets of RNA between infected and uninfected birds (*Eurasian siskins*), demonstrating shifts in response to malaria infection (60) (Table 1).

A recent study has shown that microRNA (miRNA) expression in pigs is altered by *T. gondii*. In addition, genes related to immune responses are differently regulated when compared to the splenocyte miRNA expression profiles during acute and chronic toxoplasmosis (61) (Table 1).

### Systems Vaccinology

Systems biology-based approaches have been applied in malaria research in immunology and vaccine development since the last decade. Tran and Crompton (2020) (66) have reviewed and well-documented all the information regarding this issue. It is important to highlight that a well stablished standard protocols are necessary for the assessment of vaccine efficacy for each disease to allow comparative evaluation. Studies in malaria infections have adopted a standardized safety procedure, known as controlled human malaria infection (CHMI) that has been applied, among other purposes, to the fields of malaria vaccine and drug development, supporting immunology studies (67) and systems vaccinology approaches.

Among studies carried out on malaria in systems vaccinology, it is interesting to mention that Vahey and collaborators (2010) (62) have found genes of the immunoproteasome pathway (related to the degradation of proteins for later presentation by the Major Histocompatibility Complex) that correlate with protection against *P. falciparum* infection after the third vaccination with the licensed vaccine against human malaria RTS,S, suggesting a potential role in mediating protection in vaccinated people that may represent a useful surrogate marker (Table 1). Three recent studies carried out by Van de Berg et al. (2017) (63), Kazmin et al. (2017) (64) and Tran et al. (2019) (65) have provided further insight into vaccine-mediated protection, providing molecular signatures of protective immunity against malaria (Figure 1 and Table 1). By analyzing longitudinal peripheral blood transcriptome and immunogenicity data from a clinical efficacy trial, in which healthy adults received three RTS,S doses 4 weeks apart followed by CHMI 2 weeks later, Van de Berg and collaborators (2017) (63) found that NF-κB and IFN-γ pathways may induce protection in RTS,S vaccination. On the other hand, a previous study carried out by Kazmin and collaborators (2017) (64) has suggested that multiple mechanisms can induce protective immunity against *P. falciparum* given that specific antibody titers were associated with protection in the first vaccinated group but not in the second vaccinated group, where protection was associated with polyfunctional CD4+ T cell responses. It is interesting to highlight that in this study different vaccine protocols were used, the first group of 21 volunteers received three consecutive immunizations of RTS,S/AS01 (RRR regimen), whereas in the second one the 25 volunteers received two immunizations with RTS,S/ AS01 followed by immunization with adenovirus 35 expressing CSP (ARR regimen).

Additionally, systems vaccinology has also been applied for the analyses of adjuvants (68), discerning new mechanisms of action that allow the amplification of immune responses. Hence, systems biology would allow increasing the reduced number of adjuvants that are currently in use for human vaccine formulations.

## Discussion

Next-generation sequencing (NGS) technologies have recently been applied to many apicomplexan parasites. NGS allow the reading of simultaneous millions of sequences in a short period and at a low cost per base pair. Together with multiplex platforms (RNA-seq or gene microarrays, among others), high-resolution techniques (such as AP-SMALDI MSI), and novel bioinformatic approaches, the way that biological molecules are sequenced have been revolutionized, facilitating a deeper insight into parasite-host interactions, transmission, epidemiology, and as a consequence novel therapeutic targets (Figure 1). Cutting-edge technologies such as gene editing by CRISPR-Cas9 have also allowed the discovery and functional characterization of potential novel vaccine antigens.

The combination of “omics”, computational approaches, and statistics opens the field of systems biology studies and offers the possibility to integrate the complex biology of Apicomplexa and their hosts in a holistic way. This new approach leads to a better understanding of the mechanisms used by parasites to avoid host immune defenses and by hosts to balance parasite actions, leaving behind the reductionist approach, which analyses the individual components to infer the behavior of complex systems.

New technologies such as single-cell RNA-seq platforms can overcome current limitations of “bulk measures” with high heterogeneity and analytical variability (69). Whereas the integration of other “omics” (such as proteomics, metabolomics, lipidomics, or glycomics) can facilitate and increase the number of systems biology-based studies, generating new knowledge in host-parasites interactions and immunology.

Additionally, systems biology in the context of vaccination could provide novel insights into mechanisms of action of vaccines and molecular signatures involved in protection (Figure 1) to improve design and effectiveness, providing relevant information before vaccine efficacy and safety is assessed in clinical or field trials. These integrative approaches are being incorporated into vaccine development research of protozoa parasites, beyond malarial research, which was pioneering in the field about a decade ago [recently reviewed (66)].

Nevertheless, either vaccine candidates derived from “omics”, computational approaches, or proteins with a validated a specific biological function by genetic manipulation approaches, will still require experimental validation in currently available animal models for the different diseases, evaluating vaccine-specific immune responses, immunoprotection, and safety. In consequence, well-established and unified protocols for animal models to evaluate different diseases caused by Apicomplexa parasites are paramount. Therefore, systems vaccinology combined with experimental validation and subsequent evaluation in animal models can significantly improve the novel design of vaccines against apicomplexan parasites, opening a new era of vaccinology research that could lead to an expansion in licensed products (Figure 1) after decades of significant but slow advances.

## Author Contributions

The manuscript was drafted by MT and VM-H. Tables and figures were created by MT and AR. All authors contributed to the final version.

## Funding

The Researchers were supported by the followings grants: PICT 2018-00834; INTA 2019: PD-E5-I103-001; PD-E5-I105-001; PD-E5-I102-001; Cooperative Program for the Regional Agricultural Technology Fund (FONTAGRO) Project ATN/RF-18136-RG, 2019; and financial support from the Inter-American Development Bank (BID).

## Conflict of Interest

The authors declare that the research was conducted in the absence of any commercial or financial relationships that could be construed as a potential conflict of interest.

## Publisher's Note

All claims expressed in this article are solely those of the authors and do not necessarily represent those of their affiliated organizations, or those of the publisher, the editors and the reviewers. Any product that may be evaluated in this article, or claim that may be made by its manufacturer, is not guaranteed or endorsed by the publisher.

## References

[1] Florin-Christensen M Schnittger L. Parasitic protozoa of farm animals and pets. Parasitic Protozoa of Farm Animals and Pets.Switzerland, Springer International Publishing AG. (2018) p. 438. 10.1007/978-3-319-70132-5

[2] Wasmuth J Daub J Peregrín-Alvarez JM Finney CAM Parkinson J. The origins of apicomplexan sequence innovation. Genome Res. (2009) 7:1202–13. 10.1101/gr.083386.108 19363216 PMC2704437

[3] Yuan Z-G Zhang X-X Lin R-Q Petersen E He S Yu M. Protective effect against toxoplasmosis in mice induced by DNA immunization with gene encoding Toxoplasma gondii ROP18. Vaccine. (2011) 29:6614–9. 10.1016/j.vaccine.2011.06.110 21762755

[4] Jenner E. An inquiry into the causes and effects of the variole vaccine, or cow-pox. Vaccin Against Smallpox (1798)

[5] Pasteur M. An Address on Vaccination in Relation to Chicken Cholera and Splenic Fever. Br Med J. (1881) 2:283–4. 10.1136/bmj.2.1076.283 20749974 PMC2264103

[6] Pound J. Tick fever. Notes on the inoculation of bulls as a preventive against tick fever at Rathdowney and Rosedale. Queensl Agric J. (1897) 1471–7.

[7] Connaway J Francis M. Texas fever. Experiments made by the Missouri experiment station and the Missouri state board of agriculture in cooperation with the Texas experiment station in immunizing northern breeding cattle against Texas fever for the southern trade Mo. Agric Exp Stn Bull. (1899) 48:1–64.

[8] Danforth HD. Use of live oocyst vaccines in the control of avian coccidiosis: Experimental studies and field trials. Int J Parasitol. (1998) 28:1099–109. 10.1016/S0020-7519(98)00078-2 9724881

[9] Barriga OO A. review on vaccination against protozoa and arthropods of veterinary importance. Vet Parasitol. (1994) 55:29–55. 10.1016/0304-4017(94)90054-X 7886919

[10] World Health Organization. The World malaria report 2018. Who. (2018).

[11] Laurens MB. RTS,S/AS01 vaccine (Mosquirix^TM^): an overview. Hum Vaccin Immunother. (2020) 3:480–9. 10.1080/21645515.2019.1669415 31545128 PMC7227679

[12] European Medicines Agency. First malaria vaccine receives positive scientific opinion from EMA Mosquirix to be used for vaccination of young children (2015) Available online at: http://www.ema.europa.eu/docs/en_GB/document_library/Press_release/2015/07/WC500190447.pdf (accessed Oct 20, 2021).

[13] Whorld Health Organization. WHO recommends groundbreaking malaria vaccine for children at risk (2021). Available online at: https://www.who.int/news/item/06-10-2021-who-recommends-groundbreaking-malaria-vaccine-for-children-at-risk (accessed Oct 21, 2021).

[14] Singh AK Verma AK Neha Tiwari R Karthik K Dhama K. Trends and advances in vaccines against protozoan parasites of veterinary importance: A review. J Biol Sci. (2014) 14:95–109. 10.3923/jbs.2014.95.109

[15] Blake DP Pastor-Fernández I Nolan MJ Tomley FM. Recombinant anticoccidial vaccines - a cup half full? Infect Genet Evol. (2017) 55:358–65. doi.org/10.1016/j.meegid.2017.10.009 10.1016/j.meegid.2017.10.009 29017798

[16] Liu S Wang L Zheng H Xu Z Roellig DM Li N. Comparative genomics reveals *Cyclospora cayetanensis* possesses coccidia-like metabolism and invasion components but unique surface antigens. BMC Genomics. (2016) 17:316. 10.1186/s12864-016-2632-3 27129308 PMC4851813

[17] Palmieri N Shrestha A Ruttkowski B Beck T Vogl C Tomley F. The genome of the protozoan parasite *Cystoisospora suis* and a reverse vaccinology approach to identify vaccine candidates. Int J Parasitol. (2017) 47:189–202. 10.1016/j.ijpara.2016.11.007 28161402 PMC5354109

[18] Marugan-Hernandez V Long E Blake D Crouch C Tomley F. *Eimeria tenella* protein trafficking: Differential regulation of secretion versus surface tethering during the life cycle. Sci Rep. (2017) 7:1–13. 10.1038/s41598-017-04049-1 28676667 PMC5496917

[19] Vinayak S Pawlowic MC Sateriale A Brooks CF Studstill CJ Bar-Peled Y. Genetic modification of the diarrhoeal pathogen *Cryptosporidium parvum*. Nature. (2015) 523:477–80. 10.1038/nature14651 26176919 PMC4640681

[20] Tavassoly I Goldfarb J Iyengar R. Systems biology primer: The basic methods and approaches. Essays Biochem. (2018). 10.1042/EBC20180003 30287586

[21] Germain RN. Will systems biology deliver its promise and contribute to the development of new or improved vaccines?: What really constitutes the study of “systems biology” and how might such an approach facilitate vaccine design. Cold Spring Harb Perspect Biol. (2018) 1:10a033308. 10.1101/cshperspect.a033308 29038120 PMC6071490

[22] Hagan T. Pulendran B. Will systems biology deliver its promise and contribute to the development of new or improved vaccines?: From data to understanding through systems biology. Cold Spring Harb Perspect Biol. (2018) 10:a028894. 10.1101/cshperspect.a028894 29038113 PMC5902663

[23] Alonso AM Corvi MM Diambra L. Gene target discovery with network analysis in Toxoplasma gondii. Sci Rep. (2019) 9:646. 10.1038/s41598-018-36671-y 30679502 PMC6345969

[24] Hagan T Nakaya HI Subramaniam S Pulendran B. Systems vaccinology: Enabling rational vaccine design with systems biological approaches. Vaccine. (2015) 33:5294–301. 10.1016/j.vaccine.2015.03.072 25858860 PMC4581890

[25] Gardner MJ Hall N Fung E White O Berriman M Hyman RW. Genome sequence of the human malaria parasite *Plasmodium falciparum*. Nature. (2002) 419:498–511. 10.1038/nature01097 12368864 PMC3836256

[26] Swann J Jamshidi N Lewis NE Winzeler EA. Systems analysis of host-parasite interactions. Wiley Interdiscip Rev Syst Biol Med. (2015) 7:381–400. 10.1002/wsbm.1311 26306749 PMC4679367

[27] Reid AJ Blake DP Ansari HR Billington K Browne HP Bryant J. Genomic analysis of the causative agents of coccidiosis in domestic chickens. Genome Res. (2014) 24:1676–85. 10.1101/gr.168955.113 25015382 PMC4199364

[28] Blake DP Billington KJ Copestake SL Oakes RD Quail MA Wan KL. Genetic mapping identifies novel highly protective antigens for an apicomplexan parasite. PLoS Pathog. (2011) 7:e1001279. 10.1371/journal.ppat.1001279 21347348 PMC3037358

[29] Zhang H Guo F Zhou H Zhu G. Transcriptome analysis reveals unique metabolic features in the *Cryptosporidium parvum* Oocysts associated with environmental survival and stresses. BMC Genomics. (2012) 13:647. 10.1186/1471-2164-13-647 23171372 PMC3542205

[30] Cleary MD Singh U Blader IJ Brewer JL Boothroyd JC. *Toxoplasma gondii* asexual development: Identification of developmentally regulated genes and distinct patterns of gene expression. Eukaryot Cell. (2002) 1:329–40. 10.1128/EC.1.3.329-340.2002 12455982 PMC118016

[31] McGettigan PA. Transcriptomics in the RNA-seq era. Curr Opin Chem Biol. (2013) 17:4–11. 10.1016/j.cbpa.2012.12.008 23290152

[32] Pittman KJ Aliota MT Knoll LJ. Dual transcriptional profiling of mice and *Toxoplasma gondii* during acute and chronic infection. BMC Genomics. (2014) 15:806. 10.1186/1471-2164-15-806 25240600 PMC4177681

[33] Bremner A Kim S Morris KM Nolan MJ Borowska D Wu Z. Kinetics of the cellular and transcriptomic response to eimeria maxima in relatively resistant and susceptible chicken lines. Front Immunol. (2021) 2:653085. 10.3389/fimmu.2021.653085 33841436 PMC8027475

[34] Krishna R Xia D Sanderson S Shanmugasundram A Vermont S Bernal A. A large-scale proteogenomics study of apicomplexan pathogens-*Toxoplasma gondii* and *Neospora caninum*. Proteomics. (2015) 15:2618–28. 10.1002/pmic.201400553 25867681 PMC4692086

[35] Florens L Washburn MP Raine JD Anthony RM Grainger M Haynes JD. A proteomic view of the *Plasmodium falciparum* life cycle. Nature. (2002) 419:520–6. 10.1038/nature01107 12368866

[36] Lasonder E Ishihama Y Andersen JS Vermunt AMW Pain A Sauerwein RW. Analysis of the *Plasmodium falciparum* proteome by high-accuracy mass spedrometry. Nature. (2002) 419:537–42. 10.1038/nature01111 12368870

[37] Snelling WJ Lin Q Moore JE Millar BC Tosini F Pozio E. Proteomics analysis and protein expression during sporozoite excystation of *Cryptosporidium parvum* (Coccidia, Apicomplexa). Mol Cell Proteomics. (2007) 6:346–55. 10.1074/mcp.M600372-MCP200 17124246

[38] Sanderson SJ Xia D Prieto H Yates J Heiges M Kissinger JC. Determining the protein repertoire of Cryptosporidium parvum sporozoites. Proteomics. (2008) 8:1398–414. 10.1002/pmic.200700804 18306179 PMC2770187

[39] Xia D Sanderson SJ Jones AR Prieto JH Yates JR Bromley E. The proteome of *Toxoplasma gondii*: Integration with the genome provides novel insights into gene expression and annotation. Genome Biol. (2008) 9:R116. 10.1186/gb-2008-9-7-r116 18644147 PMC2530874

[40] Bradley PJ Ward C Cheng SJ Alexander DL Coller S Coombs GH. Proteomic analysis of rhoptry organelles reveals many novel constituents for host-parasite interactions in *Toxoplasma gondii*. J Biol Chem. (2005) 280:34245–58. 10.1074/jbc.M504158200 16002398

[41] Marugán-Hernández V Álvarez-García G Tomley F Hemphill A Regidor-Cerrillo J Ortega-Mora LM. Identification of novel rhoptry proteins in *Neospora caninum* by LC/MS-MS analysis of subcellular fractions. J Proteomics. (2011) 74:629–42. 10.1016/j.jprot.2011.02.004 21315855

[42] Regidor-Cerrillo J Álvarez-García G Pastor-Fernández I Marugán-Hernández V Gómez-Bautista M Ortega-Mora LM. Proteome expression changes among virulent and attenuated *Neospora caninum* isolates. J Proteomics. (2012) 75:2306–18. 10.1016/j.jprot.2012.01.039 22343075

[43] Pastor-Fernández I Regidor-Cerrillo J Jiménez-Ruiz E Álvarez-Garciá G Marugán-Hernández V Hemphill A. Characterization of the *Neospora caninum* NcROP40 and NcROP2Fam-1 rhoptry proteins during the tachyzoite lytic cycle. Parasitology. (2015) 143:97–113. 10.1017/S0031182015001511 26521890

[44] Kumar V Ray S Aggarwal S Biswas D Jadhav M Yadav R. Multiplexed quantitative proteomics provides mechanistic cues for malaria severity and complexity. Commun Biol. (2020) 3:683. 10.1038/s42003-020-01384-4 33204009 PMC7672109

[45] Welti R Mui E Sparks A Wernimont S Isaac G Kirisits M. Lipidomic Analysis of *Toxoplasma gondii* Reveals Unusual Polar Lipids. Biochemistry. (2007) 46:13882–90. 46(48):13882–90. 10.1021/bi7011993 17988103 PMC2576749

[46] Kadesch P Hollubarsch T Gerbig S Schneider L Silva LMR Hermosilla C. Intracellular Parasites Toxoplasma gondii and Besnoitia besnoiti, Unveiled in Single Host Cells Using AP-SMALDI MS Imaging. J Am Soc Mass Spectrom. (2020) 31:1815–24. 10.1021/jasms.0c00043 32830963

[47] Bryant JM Baumgarten S Glover L Hutchinson S. Rachidi N. CRISPR in parasitology: not exactly cut and dried! Trends Parasitol. (2019) 35:409–22. 10.1016/j.pt.2019.03.004 31006600

[48] Wagner JC Platt RJ Goldfless SJ Zhang F Niles JC. Efficient CRISPR-Cas9-mediated genome editing in Plasmodium falciparum. Nat Methods. (2014) 11:915–8. 10.1038/nmeth.3063 25108687 PMC4199390

[49] Ghorbal M Gorman M MacPherson CR Martins RM Scherf A Lopez-Rubio JJ. Genome editing in the human malaria parasite *Plasmodium falciparum* using the CRISPR-Cas9 system. Nat Biotechnol. (2014) 32:819–21. 10.1038/nbt.2925 24880488

[50] Beneke T Madden R Makin L Valli J Sunter J Gluenz E. Cas9 high-throughput genome editing toolkit for kinetoplastids. R Soc Open Sci. (2017) 4:170095. 10.1098/rsos.170095 28573017 PMC5451818

[51] Zhang C Xiao B Jiang Y Zhao Y Li Z Gao H. Efficient editing of malaria parasite genome using the CRISPR/Cas9 system. MBio. (2014) 5:e01414–14. 10.1128/mBio.01414-14 24987097 PMC4161241

[52] Sidik SM Huet D Ganesan SM Huynh MH Wang T Nasamu AS. A Genome-wide CRISPR screen in *toxoplasma* identifies essential apicomplexan genes. Cell. (2016) 166:1423–35.e12. 10.1016/j.cell.2016.08.019 27594426 PMC5017925

[53] Tang X Suo J Liang L Duan C Hu D Gu X. Genetic modification of the protozoan *Eimeria tenella* using the CRISPR/Cas9 system. Vet Res. (2020) 51:4–8. 10.1186/s13567-020-00766-0 32160917 PMC7065449

[54] Sateriale A Pawlowic M Vinayak S Brooks C Striepen B. Genetic Manipulation of *Cryptosporidium parvum* with CRISPR/Cas9. In: Mead JR Arrowood MJ editors. Cryptosporidium: Methods and Protocols. New York, NY: Springer New York (2020). p. 219–28. 10.1007/978-1-4939-9748-0_13 31452165

[55] Young J Dominicus C Wagener J Butterworth S Ye X Kelly G. A CRISPR platform for targeted in vivo screens identifies *Toxoplasma gondii* virulence factors in mice. Nat Commun. (2019) 10:3963. 10.1038/s41467-019-11855-w 31481656 PMC6722137

[56] Gas-Pascual E Ichikawa HT Sheikh MO Serji MI Deng B Mandalasi M. CRISPR/Cas9 and glycomics tools for *Toxoplasma* glycobiology. J Biol Chem. (2019) 294:1104–25. 10.1074/jbc.RA118.006072 30463938 PMC6349120

[57] Ockenhouse CF Hu WC Kester KE Cummings JF Stewart A Heppner DG. Common and divergent immune response signaling pathways discovered in peripheral blood mononuclear cell gene expression patterns in presymptomatic and clinically apparent malaria. Infect Immun. (2006) 74:5561–73. 10.1128/IAI.00408-06 16988231 PMC1594921

[58] Colborn JM Ylöstalo JH Koita OA Cissé OH Krogstad DJ. Human Gene Expression in Uncomplicated *Plasmodium falciparum* Malaria. J Immunol Res. (2015) 2015:162639. 10.1155/2015/162639 26491700 PMC4605373

[59] Yamagishi J Natori A Tolba MEM Mongan AE Sugimoto C Katayama T. Interactive transcriptome analysis of malaria patients and infecting *Plasmodium falciparum*. Genome Res. (2014) 24:1433–44. 10.1101/gr.158980.113 25091627 PMC4158759

[60] Videvall E Cornwallis CK Palinauskas V Valkiunas G Hellgren O. The Avian Transcriptome Response to Malaria Infection. Mol Biol Evol. (2015) 32:1255–67. 10.1093/molbev/msv016 25636457 PMC4408411

[61] Hou Z Liu D Su S Wang L Zhao Z Ma Y. Comparison of splenocyte microRNA expression profiles of pigs during acute and chronic toxoplasmosis. BMC Genomics. (2019) 20:97. 10.1186/s12864-019-5458-y 30700253 PMC6354428

[62] Vahey MT Wang Z Kester KE Cummings J Heppner DG Nau ME. Expression of genes associated with immunoproteasome processing of major histocompatibility complex peptides is indicative of protection with adjuvanted RTS,S malaria vaccine. J Infect Dis. (2010) 201:580–9. 10.1086/650310 20078211

[63] van den Berg RA Coccia M Ballou WR Kester KE Ockenhouse CF Vekemans J. Predicting RTS,S vaccine-mediated protection from transcriptomes in a malaria-challenge clinical trial. Front Immunol. (2017) 8:557. 10.3389/fimmu.2017.00557 28588574 PMC5440508

[64] Kazmin D Nakaya HI Lee EK Johnson MJ van der Most R van den Berg RA. Systems analysis of protective immune responses to RTS,S malaria vaccination in humans. Proc Natl Acad Sci. (2017) 114:2425–30. 10.1073/pnas.1621489114 28193898 PMC5338562

[65] Tran TM Bijker EM Haks MC Ottenhoff THM Visser L Schats R. Whole-blood transcriptomic signatures induced during immunization by chloroquine prophylaxis and *Plasmodium falciparum* sporozoites. Sci Rep. (2019) 9:8386. 10.1038/s41598-019-44924-7 31182757 PMC6557840

[66] Tran TM Crompton PD. Decoding the complexities of human malaria through systems immunology. Immunol Rev. (2020) 293:144–62. 10.1111/imr.12817 31680289 PMC6944763

[67] Stanisic DI McCarthy JS Good MF. Controlled human malaria infection: Applications, advances, and challenges. Infect Immun. (2018) 86:e00479–17. 10.1128/IAI.00479-17 28923897 PMC5736798

[68] Harandi AM. Systems analysis of human vaccine adjuvants. Semin Immunol. (2018) 39:30–4. 10.1016/j.smim.2018.08.001 30122362

[69] Papalexi E Satija R. Single-cell RNA sequencing to explore immune cell heterogeneity. Nature Reviews Immunology. (2018) 18:35–45. 10.1038/nri.2017.76 28787399

